# Two Cases of Remimazolam Anesthesia Managed With Pharmacokinetic Simulations in an Awake Craniotomy of Patients With Obesity

**DOI:** 10.7759/cureus.69311

**Published:** 2024-09-13

**Authors:** Takehito Sato, Kimitoshi Nishiwaki

**Affiliations:** 1 Anesthesiology, Nagoya University Hospital, Nagoya, JPN; 2 Anesthesiology, Nagoya University Graduate School of Medicine, Nagoya, JPN

**Keywords:** anesthesia, awake craniotomy, brain tumor, obesity, remimazolam

## Abstract

Anesthetic management for awake craniotomy (AC) often poses problems in patients with obesity including respiratory management. The Japan Awake Craniotomy Guidelines state that the indication for surgery should be carefully considered, especially in patients with obesity (body mass index (BMI) > 30). Patients with obesity often have comorbidities such as dyslipidemia, hypertension, and type 2 diabetes, and this is a risk factor for perioperative morbidity and mortality.

Remimazolam, a new intravenous anesthetic, has been reported to be useful in anesthesia management during AC, but its use in obese patients has not been reported. Herein, we report two cases case series in which remimazolam was used in patients with obesity and safely managed under anesthesia with the pharmacokinetic simulations.

## Introduction

Anesthetic management for awake craniotomy (AC) poses challenges due to respiratory considerations, particularly in patients with obesity. The Japan Awake Craniotomy Guidelines emphasize careful consideration of surgical indications, especially in patients with obesity (body mass index (BMI) > 30) [1].

Remimazolam, a short-acting benzodiazepine agent has demonstrated utility in anesthesia management during AC [2,3]. Previous studies have shown that the combination of remimazolam and flumazenil results in faster awakening times compared to propofol during AC. The combination of remimazolam and flumazenil may result in faster awakening than propofol and suggests that the combination of remimazolam and flumazenil may be a useful anesthetic for the asleep-awake-asleep AC method [2,3].

However, its application in patients with obesity remains unexplored. Here, we present our first case experience utilizing remimazolam in patients with obesity with a BMI of 37 and 32, which were safely managed under anesthesia with pharmacokinetic simulations in using the Schüttler model [4].

## Case presentation

Case 1

**Figure 1:**
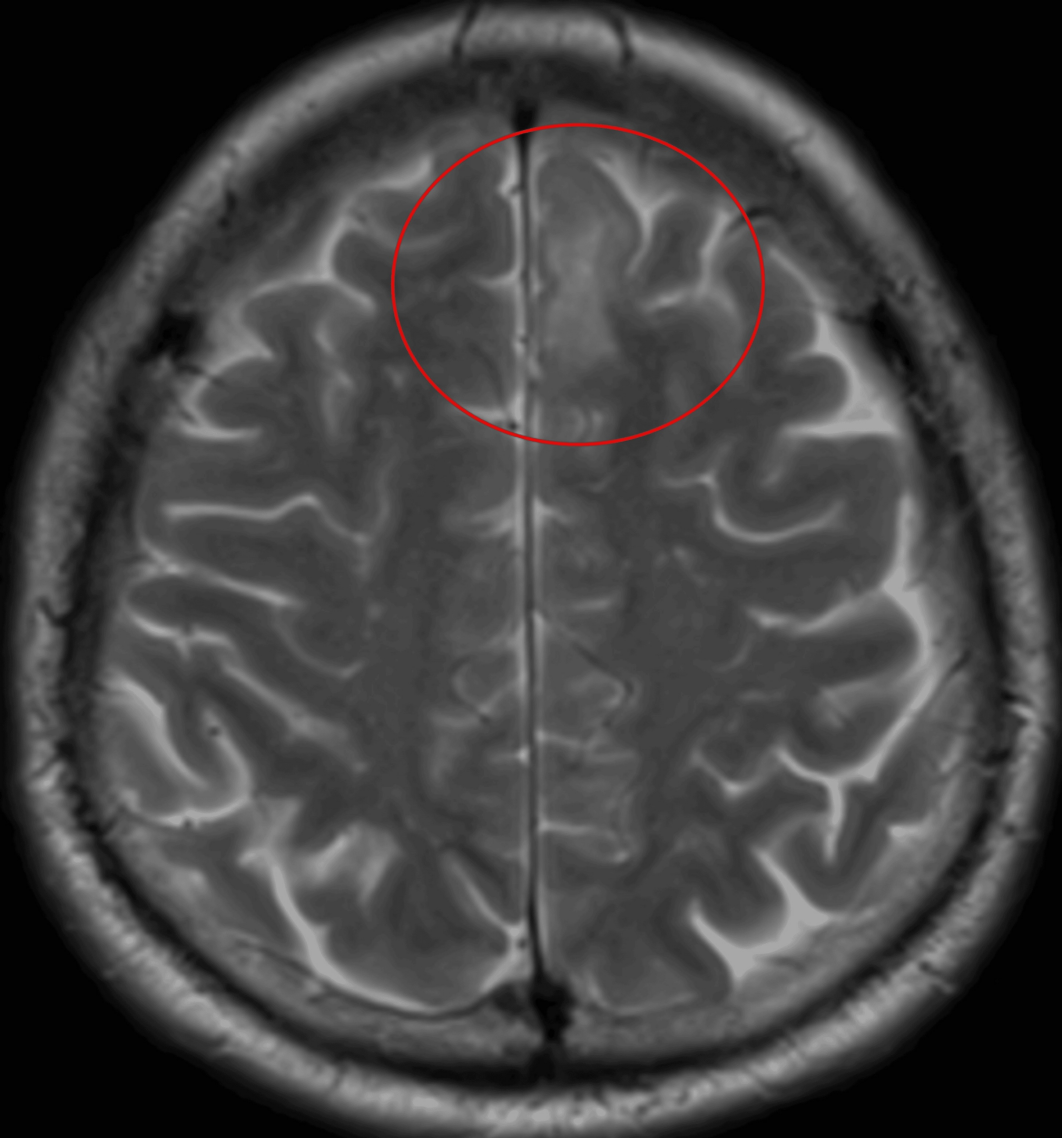
MRI revealed a tumorous lesion in the right frontal lobe.

The patient was a 44-year-old male. He was 163 cm tall and weighed 98.4 kg (BMI, 37.4). His medical history included diabetes mellitus (DM) and obstructive sleep apnea (OAS). He was diagnosed with a left frontal lobe tumor and was scheduled for elective AC (Figure 1).

Remimazolam (Anerem® 50 mg; Mundipharma K.K., Tokyo, Japan) was administered at 12 mg/kg/h (calculated by actual body weight) and remifentanil was administered at 0.1 μg/kg/min (by ideal body weight), inducing loss of consciousness within 102 s (effect-site concentration: 4.01 μg/mL).

A supraglottic airway device (I-gel # 5 Intersurgical, Wokingham, Berkshire, UK) was inserted after confirming the loss of consciousness, and mechanical ventilation was initiated. Vital signs were recorded using non-invasive and invasive blood pressure monitoring, capnography, electrocardiography, and pulse oximetry (SpO2). After inserting the arterial and urinary catheters, bilateral scalp blocks (supraorbital and supratrochlear, greater and lesser occipital, auriculotemporal, and zygomaticotemporal branch nerves) were performed with 0.375% ropivacaine and 1:200,000 adrenaline to reduce intraoperative pain [2-4]. Anesthetic maintenance comprised remimazolam at 0.5-0.6 mg/kg/h and remifentanil at 0.1-0.15 μg/kg/min with an effect-site concentration of approximately 0.65 μg/mL during maintenance.

Bispectral index (BIS) values remained between 40 and 50 before the awake phase. Approximately 10 min after discontinuing anesthetic administration, spontaneous breathing recovered (effect-site concentration: 0.33 μg/mL), followed by signs of arousal such as body movement and eye-opening, prompting the removal of the supraglottic airway (SGA) 15 min post-discontinuation (effect-site concentration: 0.27 μg/mL). Despite not receiving flumazenil, a clear awakening ensued. The patient performed intraoperative tasks without any complications, and no complications such as hypoxemia were observed. Awake time was 97 min. General anesthesia was reintroduced with remimazolam, and the AC was completed without any complications.

Case 2

**Figure 2:**
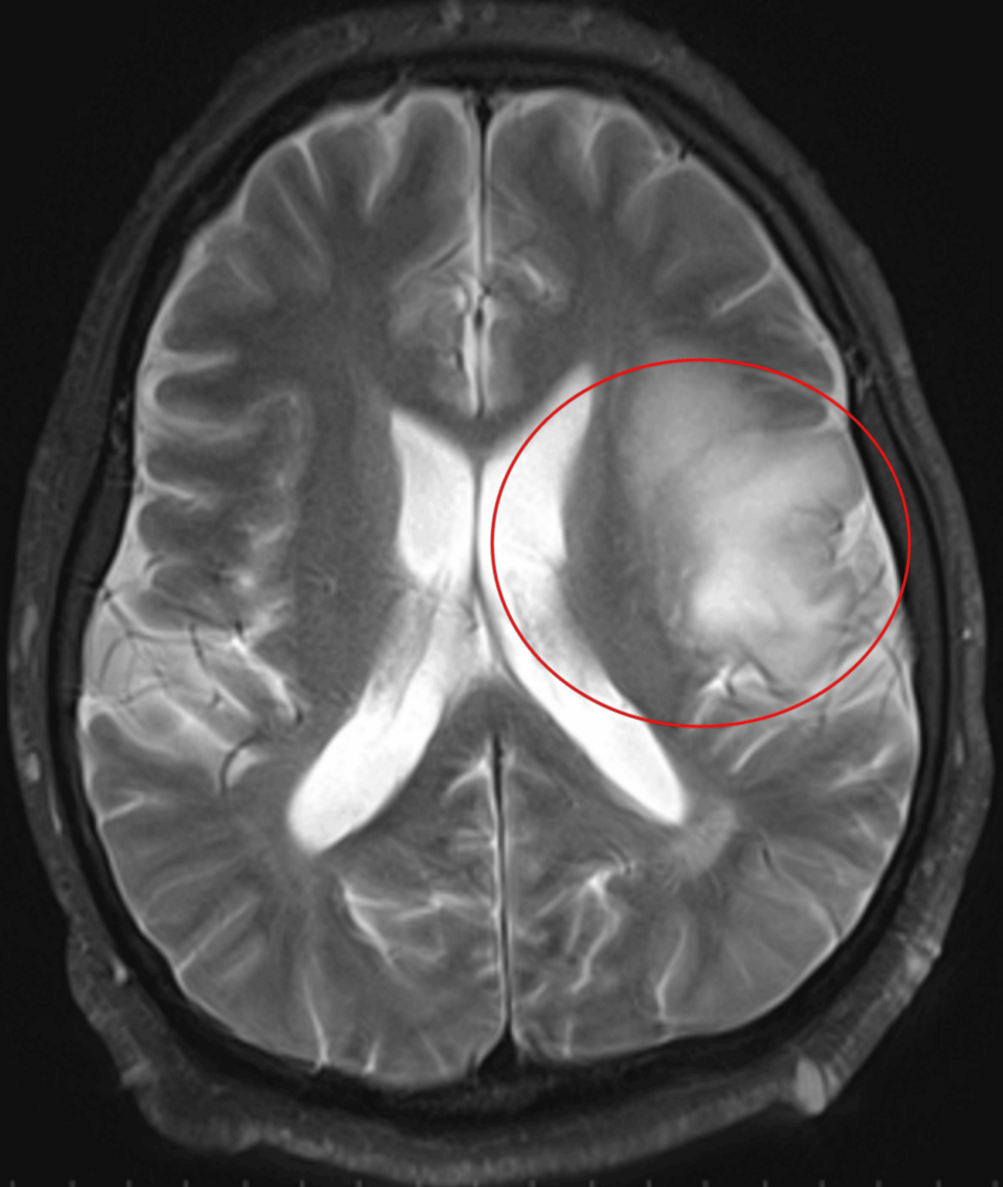
MRI revealed a high intensity area in the left insular gyrus

The patient was a 54-year-old male. He was 168 cm tall and weighed 90.7 kg (BMI: 32). His medical history included neurofibromatosis type 2 and OAS. He was diagnosed with a left insula tumor and was scheduled for elective AC (Figure 2).

Remimazolam (Anerem® 50 mg; Mundipharma K.K., Tokyo, Japan) was administered at 12 mg/kg/h (calculated by actual body weight) and Remifentanil at 0.15 μg/kg/min, inducing loss of consciousness within 95 s (effect-site concentration: 3.88 μg/mL).

After confirming the loss of consciousness, a supraglottic airway device (Igel # 4 Intersurgical, Wokingham, Berkshire, UK) was inserted, and mechanical ventilation was initiated. Bilateral scalp blocks were subsequently performed. Anesthetic maintenance comprised remimazolam at 0.5-0.6 mg/kg/h and Remifentanil at 0.1-0.15 μg/kg/min with an effect-site concentration of approximately 0.78 μg/mL during maintenance. The BIS values remained between 40 and 60 before the awake phase. Approximately 12 min after discontinuing anesthetic administration, spontaneous breathing recovered (effect-site concentration: 0.33 μg/mL), followed by signs of arousal such as body movement and eye-opening, prompting the removal of the Igel 15 min post-discontinuation (effect-site concentration: 0.28 μg/mL) after administered 0.5 mg of flumazenil.

The patient performed intraoperative tasks without any complications, and no complications such as hypoxemia were observed. Awake time was 180 min. General anesthesia was reintroduced with remimazolam, and the AC was completed without any complications. Written informed consent was obtained from patients for this case report.

## Discussion

The obesity epidemic has grown to epic proportions. Patients with obesity are considered to be at higher risk of perioperative complications including anesthetic complications. Anesthesiologists have to manage unique challenges when treating patients with obesity, including difficulties with venous access, endotracheal intubation, risk of obesity-related complications, and risk of postoperative complications [5].

Patients with obesity often have comorbidities such as dyslipidemia, hypertension, and type 2 diabetes, which is also known as metabolic syndrome. Metabolic syndrome is an independent predictor of cardiac dysfunction and cardiovascular disease, and a risk factor for perioperative morbidity and mortality [5]. In 2015, the Society of Anesthesiologists of Great Britain and Ireland Society of Obesity and Bariatric Anesthesiology published guidelines for the perioperative care of patients with obesity [6]. The guidelines include recommendations for anesthesia care specifically for patients with obesity and recommend that central obesity and metabolic syndrome be part of perioperative risk stratification.

Remimazolam is an ultra-short-acting benzodiazepine anesthetic, and its safety has been reported for intraoperative management for AC, providing anesthesia management comparable to propofol [2,3]. Our case showed that remimazolam could be safely used in patients with obesity, suggesting its potential benefit to this high-risk demographic. However, it has been reported that the blood concentration of remimazolam may be higher in patients with obesity, depending on their actual body weight. The optimal blood concentration of remimazolam during general anesthesia maintenance reportedly averages 0.69±0.23 μg/mL [4,7]. Therefore, we managed anesthesia using pharmacokinetic simulations to avoid overdose. Consequently, AC could be performed without delaying arousal and agitation. In contrast, a previous study suggested that the calculation of maintenance doses should be based on the adjusted body weight (ABW), ideal body weight (IBW), and total body weight (TBW) derived from the formula [7,8]:

IBW (kg)=45.4+0.89×(Height−152.4)+4.5×(1−Sex)

ABW (kg)=IBW+0.4×(TBW−IBW)

“Sex” is 0 for males and 1 for females, and “Height” is calculated in centimeters.

Therefore, to prevent remimazolam overdose, it is very important to use ABW instead of actual body weight or, use pharmacokinetic simulation as in this case. Furthermore, in this case, clear awakening was achieved by using an antagonist of remimazolam in Case 2. This case series revealed that the use of remimazolam as an anesthetic agent in AC was comparable to the use of propofol. This effect was achieved by antagonizing remimazolam with flumazenil, resulting in faster awakening and better intraoperative task achievement). The antagonistic effect of flumazenil on remimazolam anesthesia in patients with obesity undergoing AC remains controversial as no reports have been published yet. It is possible that administration of flumazenil to these patients may result in a clearer awakening, but further research is needed.

## Conclusions

 In conclusion, we report a case in which remimazolam was safely administered in combination with a pharmacokinetic simulations for AC in the patients with obesity without any intraoperative complications.By using a pharmacokinetic simulations during the surgery, we were able to prevent remimazolam overdose and manage the anesthesia at an optimal depth. And as a result, AC could be performed without delaying awakening and arousal.We have concluded that remimazolam besylate could be used for intraoperative anesthetic management of AC in the patients with obesity.
